# Clinical outcome and prognostic factors of patients with non-traumatic angiography-negative subarachnoid hemorrhage

**DOI:** 10.3389/fneur.2023.1157845

**Published:** 2023-07-20

**Authors:** Yibo Geng, Jianwen Jia, Xiaoli Liu, Tong Li, He Liu, Yongquan Sun, Yang Wang

**Affiliations:** ^1^Department of Neurosurgery, Beijing Chaoyang Hospital, Capital Medical University, Beijing, China; ^2^Department of Hernia and Abdominal Wall Surgery, Beijing Chaoyang Hospital, Capital Medical University, Beijing, China

**Keywords:** negative angiography, non-traumatic subarachnoid hemorrhage, non-perimesencephalic, modified Rankin scale, Hijdra cistern score, risk factor

## Abstract

**Purpose:**

The cause of spontaneous subarachnoid hemorrhage (SAH) is unknown in 10% of cases. The aim of this study was to demonstrate the characteristics of patients with angiography-negative subarachnoid hemorrhage (anSAH) and to analyze factors influencing the clinical outcome in patients suffering from anSAH.

**Methods:**

A retrospective cohort of 75 patients with anSAH [26 perimesencephalic (pmSAH) and 49 non-perimesencephalic SAH (npmSAH)] admitted between January 2016 and June 2022 was included. We analyzed demographic, clinical data and 6-month functional outcomes. Enter regression analysis was performed to identify factors associated with outcomes.

**Results:**

Unfavorable outcome was achieved in 10 of 75 patients (13.3%). Unfavorable outcome was associated with senior adults (*p* = 0.008), Hijdra cistern score (HCS) elevation (*p* = 0.015), long-time lumbar cistern continuous drainage (LCFD; *p* = 0.029) and hydrocephalus (*p* = 0.046). The only significant risk factor for unfavorable outcome after npmSAH was the HCS (*OR* 1.213 (95%CI 1.007–1.462), *p* = 0.042).

**Conclusion:**

Our study provides valuable information on both SAH patterns and functional outcome in patients suffering from anSAH and should be taken into consideration during management of these patients.

## 1. Introduction

**Figure 1:**
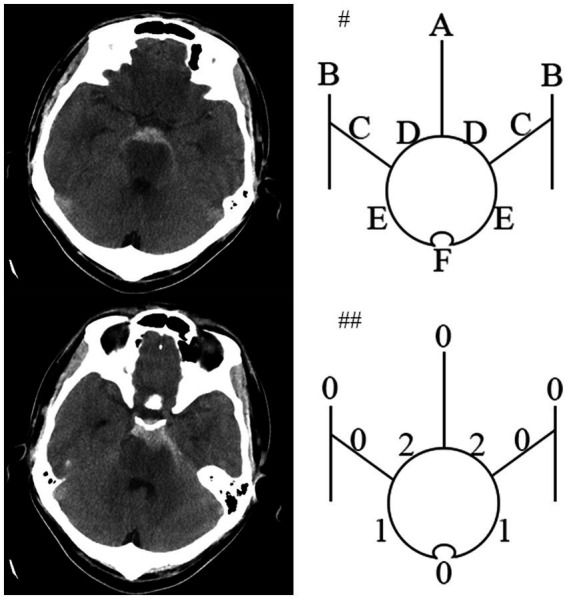
An pmSAH example of the HCS developed by Hijdra et al. Computed tomogram of a 34-year-old patient around 18 hours after pmSAH on left side. #Top diagram identifies 10 basal cisterns and fissures: A, frontal interhemispheric fissure; B, sylvian fissure, lateral parts; C, sylvian fissure, basal parts; D, Suprasellar cistern; E, ambient cisterns; F, quadrigeminal cistern. ##The bottom diagram indicates the amount of blood in each cistern and fissure. HCS is 6 points (Modified from Hijdra (12)).

**Figure 2:**
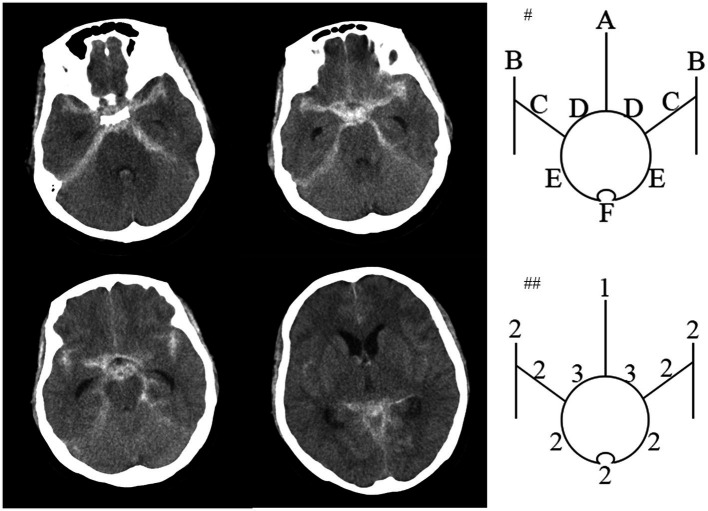
An npmSAH example of the HCS. Computed tomogram of a 62-year-old patient around 4 hours after npmSAH on left side. #Top diagram identifies 10 basal cisterns and fissures: A, frontal interhemispheric fissure; B, sylvian fissure, lateral parts; C, sylvian fissure, basal parts; D, Suprasellar cistern; E, ambient cisterns; F, quadrigeminal cistern. ##The bottom diagram indicates the amount of blood in each cistern and fissure. HCS is 21 points.

Spontaneous subarachnoid hemorrhage (SAH) is usually caused by the rupture of an intracranial aneurysm and other rare diseases included Moyamoya disease, arteriovenous malformation, neoplasm and cortical thrombosis. However, approximately 10% of SAH patients could not identify the proper bleeding source, although repeated radiological imaging was performed, which was called angiography-negative SAH (anSAH) (1, 2). It is well-understand that anSAH is generally considered to follow a significantly more benign course with a better prognosis compared to aneurysmal SAH (3). anSAH consists of pmSAH (Figure 1) and npmSAH, or diffuse SAH (Figure 2), depending on distinct bleeding distribution patterns. The risk factor for outcome in pmSAH patients has been investigated by several studies (4, 5). However, data on patients suffering from npmSAH is limited. There are a few studies reporting on a limited number of patients (6–9). The present study focused on the characteristics and clinical outcomes of anSAH, especially npmSAH.

## 2. Methods

### 2.1. Patient selection

Consecutive patients with non-traumatic SAH admitted between January 2016 and June 2022 with negative angiography were retrospectively enrolled. Those with symptom onset over 24 h, an aneurysm diagnosed on subsequent computed tomography angiography, magnetic resonance angiography or angiography and missing follow-up were excluded. Finally, 75 anSAH patients were included in our study. The study was approved by the Beijing Chaoyang Hospital Ethics Committee.

### 2.2. Imaging protocol

In our institution, all patients with spontaneous SAH detected by NCCT in emergency would undergo three-dimension digital subtraction angiography (DSA) of at least four vessels to rule out intracranial bleeding sources within 24 h. Femoral arterial access was performed routinely. An additional DSA was performed after 7 days to exclude an obscured vascular pathology. For the patients refusing repeated DSA, MRA or CTA was applied to confirm aneurysm free.

### 2.3. Imaging analysis

At least two senior interventional neurosurgeons analyze DSA and diagnosed anSAH. anSAH was defined as both neurosurgeons diagnosed no SAH-relating macrovascular disease after DSA. All cases were classified in accordance with the bleeding pattern (perimesencephalic or non-perimesencephalic SAH, pmSAH or npmSAH) and the modified Fisher grade (1 to 4). HCS was performed by two independent members of the research team blinded to each other’s ratings and to the participant’s outcome. Scores from the 2 members were then averaged. The definition of pmSAH modified Fisher scale (mFS) grade and HCS was mentioned previously (10–12). In brief, the criteria for pmSAH were: the SAH epicenter was immediately anterior to the midbrain and/or pon; it is acceptable that the SAH extended to the posterior part of the anterior interhemispheric fissure and the basal part of the Sylvian fissure; it is acceptable that a small amounts of sedimented blood located in the lateral ventricle occipital horn and fourth ventricle; it is acceptable that a little blood extended to the cisterna magna. The rest of anSAH patients was defined as npmSAH. For instance, the HCS was calculated as the representative figures (Figures 1, 2).

### 2.4. Data collection

All demographic and clinical data were collected by electrical medical records. CT was performed within 24 h of onset. Blood samples within 24 h of admission were achieved. The weight and height of parts of disabled patients were obtained during follow-up. Normal BMI was defined as 18.5~25 according to World Health Organization (WHO) guideline.^1^ Age on admission more than 65 were identified as senior adults. mRS at 6 months of admission was accessed by a neurological specialist by telephone or face-to-face interview, and mRS at 6 months ranging from 0 to 2 was defined as a favorable outcome.

### 2.5. Statistics

Data analyses were performed using the computer software package SPSS (V23.0). Normally distributed data are presented as mean ± SD and non-normally distributed data as median and interquartile range (IQR). Differences between two groups were analyzed using the two-sided Student’s *t*-test or Mann–Whitney *U*-test. Categorical variables were analyzed in contingency tables using the Chi-square test. The variables identified in univariate analysis (*p* < 0.05) plus essential elements for prognosis defined by senior neurosurgeons were then included in multiple logistic regression analysis with “enter” selection to determine independent predictors for 6-month unfavorable outcome. Results with *p* < 0.05 were considered statistically significant.

## 3. Results

### 3.1. Baseline population demographics between pmSAH and npmSAH

Seventy five patients developed anSAH since 2016. Patient characteristics including sex, age, medical history, SAH pattern, radiological features and treatment are summarized in Table 1. Overall, 26 (34.7%) patients suffered from pmSAH on the initial CT scan, while 49 (65.3%) were diagnosed as npmSAH. Expectedly, the HCS was lower in the pmSAH than npmSAH group (5.5 (3–7.25) vs. 12 (4.5–16), *p* < 0.001). Hunt and Hess scores on admission ranging from 3 to 4 were found in 8.0% of patients. On the aspect of radiology, the mFS score of 3 and 4 were found in 45.3% and 8.0% of patients, respectively, and with a predominant npmSAH majority. Additionally, 18 of 75 patients received LCFD to prevent CSF clogged by bleeding and the drainage duration did not show any difference between distinct SAH patterns.

**Table 1:** Comparison of possible demographic and clinical parameters of patients between different SAH patterns.

Variables	Total	Perimesencephalic	Non-perimesencephalic	*P*-value
Number of patients (*n*, %)	75 (100)	26 (34.7)	49 (65.3)	NA
Male (*n*, %)	34 (45.3)	14 (53.8)	20 (40.8)	0.281
Senior adult (*n*, %)	22 (29.3)	6 (23.1)	16 (32.7)	0.386
Smoking (*n*, %)	19 (25.3)	10 (38.5)	9 (18.4)	0.057
Hypertension (*n*, %)	32 (42.7)	15 (57.7)	17 (34.7)	0.055
Anticoagulation (*n*, %)	15 (20.0)	6 (23.1)	9 (18.4)	0.627
Cholesterol (mean, SD)	4.7 (1.0)	4.5 (1.1)	4.8 (0.9)	0.291
Abnormal BMI (*n*, %)	26 (34.7)	8 (30.8)	18 (36.7)	0.605
High NLR (*n*, %)	38 (50.7)	12 (46.2)	26 (53.1)	0.569
Admission HH 3–5 (*n*, %)	6 (8.0)	1 (3.8)	5 (10.2)	0.658
mFS 3–4 (*n*, %)	41 (54.7)	10 (38.5)	31 (63.3)	**0.04**
HCS (median, IQR)	8 (4–14)	5.5 (3.0–7.25)	12 (4.5–16.0)	**<0.001**
LCFD (mean, SD)^*^	99.8 (32.4)	87.8 (10.9)	103.2 (35.9)	0.417
Extension to ventricular (*n*, %)	11 (14.7)	2 (7.7)	9 (18.4)	0.214
Fee (kilo-RMB, mean, SD)	20.4 (9.8)	20.9 (8.3)	20.1 (10.6)	0.74

SAH, Subarachnoid hemorrhage; NA, Not applicable; BMI, Body mass index; NLR, Neutrophil-to-lymphocyte ratio; HH, Hunt and Hess score; mFS, modified Fisher scale; HCS, Hijdra cistern score; IQR, Interquartile range; LCFD, Lumbar cistern continuous drainage; ^*^18 patients performed LCFD; RMB, Chinese yuan. Bold values mean *P* < 0.05.

### 3.2. Factors influencing functional outcome of anSAH

Overall, 65 (86.7%) anSAH patients achieved favorable outcome (Table 2). Firstly, Patients with unfavorable outcome were significantly more common in senior adults than others (*p* = 0.008). Next, Patients with hydrocephalus on admission were another risk factor for poor prognosis (*p* = 0.046). In addition, patients with unfavorable outcome showed longer CSF drainage period (119.5 ± 18.5 vs. 89.9 ± 33.9, *p* = 0.029) and higher HCS [14 (7–20) vs. 7 (3–13), *p* = 0.029]. Patients with intraventricular hematoma tended to be more common in the unfavorable outcome group (*p* = 0.051).

**Table 2:** Functional outcome in patients with anSAH.

Variables	Total	Favorable outcome	Unfavorable outcome	*P*-value
Number of patients (*n*, %)	75 (100.0)	65 (86.7)	10 (13.3)	NA
Male (*n*, %)	34 (45.3)	30 (46.2)	4 (40.0)	0.982
Senior adult (*n*, %)	22 (29.3)	15 (23.1)	7 (70.0)	**0.008**
Smoking (*n*, %)	19 (25.3)	18 (27.7)	1 (10.0)	0.42
Hypertension (*n*, %)	32 (42.7)	26 (40.0)	6 (60.0)	0.397
Anticoagulation (*n*, %)	15 (20.0)	11 (16.9)	4 (40.0)	0.203
Cholesterol (mean, SD)	4.7 (1.0)	4.6 (1.0)	4.8 (0.8)	0.597
Abnormal BMI (*n*, %)	26 (34.7)	19 (29.2)	6 (60.0)	0.118
High NLR (*n*, %)	38 (50.7)	32 (49.2)	6 (60.0)	0.768
Admission HH 3–5 (*n*, %)	6 (8.0)	4 (6.2)	2 (20.0)	0.18
mFS 3–4 (*n*, %)	41 (54.7)	34 (52.3)	7 (70.0)	0.481
HCS (median, IQR)	8 (4–14)	7 (3–13)	14 (7–20)	**0.015**
LCFD (mean, SD)^*^	99.8 (32.4)	89.9 (33.9)	119.5 (18.5)	**0.029**
Extension to ventricular (*n*, %)	11 (14.7)	7 (10.8)	4 (40.0)	0.051
pmSAH (*n*, %)	26 (34.7)	24 (36.9)	2 (20.0)	0.49
Hydrocephalus (*n*, %)	7 (9.3)	4 (6.2)	3 (30.0)	**0.046**
Alcohol addiction (*n*, %)	14 (18.7)	13 (20.0)	1 (10.0)	0.749
Diabetes mellitus (*n*, %)	16 (21.3)	12 (18.5)	4 (40.0)	0.257

* 18 patients performed LCFD. Bold values mean *P* < 0.05.

To rule out the confounders between both SAH patterns, we analyzed the two distinct anSAH patterns separately (Supplementary Table 1). As expected, the HCS was a unique variable, which elevation associated with unfavorable outcome in npmSAH [16 (9.5–22) vs. 11 (3.5–11.5), *p* = 0.032], instead of pmSAH (*p* = 0.615).

### 3.3. HCS predicted the neurological outcome

Finally, the multivariate logistic analysis with enter selection method was performed and the results revealed that a high HCS was an independent risk factor for outcome in npmSAH patients (*p* = 0.042, *OR* = 1.213, 95%CI: 1.007–1.462; Table 3).

**Table 3:** Multivariable logistic regression analysis for evaluating 6-month functional recovery in patients with npmSAH.

Variable	Odd ratio (95% CI)	*P*-value
Female	1.486 (0.208–10.61)	0.693
HCS	1.213 (1.007–1.462)	**0.042**
Senior adult	5.442 (0.759–39.013)	0.092
Abnormal BMI	6.757 (0.743–61.472)	0.09
Hydrocephalus	1.955 (0.233–16.422)	0.537

CI, Confidential interval. Bold value mean *P* < 0.05.

## 4. Discussion

In the study, we focused on the both distinct clinical characteristics of pmSAH and npmSAH and the risk factors of anSAH via a single-center retrospective cohort analysis. We found that age more than 65, long LCFD and hydrocephalus were associated with unfavorable outcome. Furthermore, to our knowledge, we first brought up that the HCS was a potential risk factor for unfavorable outcome after non-traumatic angiography-negative SAH patients, especially for npmSAH.

pmSAH was defined as hemorrhage restricted to the cisterns surrounding the brainstem and suprasellar cistern and presented a negative DSA result (10). Despite the number of patients with pmSAH, the etiology remains doubtable. Several studies speculated that deep venous system may be involved in pmSAH, and a rupture of the basal vein of Rosenthal around the midbrain was one of the etiologies of pmSAH (13, 14). The rupture might be caused by Valsalva maneuver, such as defecation, heavy lifting and swimming, which caused intrathoracic pressure elevation to block the internal jugular venous return, resulting in intracranial venous hypertension and leading to deep vein breakdown (15). Also, Laukka et al. suggested that physical exertion triggered pmSAH and the risk was five times higher than npmSAH (16). As the pmSAH has an extremely low incidence of hydrocephalus and intracranial vasospasm, both the survival rate and neurological function were excellent, with a 98.6% survival rate at 30 months and more than 94% favorable outcome at follow-up (17–19). In addition, several studies recommended that pmSAH was not required to perform a second angiography (20). In our research, we did not observe any death or positive DSA results in pmSAH and 8.3% (2/24) with an unfavorable outcome (both 3 mRS scores) at 6 months after onset. The two patients were both senior adults and complicated hypertension and diabetes, and suffered from thick cisternal clot, which were risk factors for SAH patients.

On the other hand, the etiology, treatment and prognosis of npmSAH were more heterogeneous compared to pmSAH. Except for the aneurysm, over half of npmSAH patients presented jugular stenosis, which had a strong effect on intravenous pressure (15). The anatomical variation plus the risk factors of venous hypertension provoked venous rupture. Nonetheless, 2%–12% of npmSAH were diagnosed as aneurysm, which had false-negative initial DSA (7, 21). Thus, a repeat DSA or CTA/MRA was mandatory for npmSAH and all patients in our project performed at least one-time repeated imaging examination (20). It was reported that approximately half of npmSAH suffered acute hydrocephalus and one-fifth indicated vasospasm, which resulted in 11%–41% of npmSAH achieving favorable outcome at three or 6-month follow-up (3, 22). In our study, 16.3% (8/49) of npmSAH were assessed unfavorable recovery at 6-month onset, which was similar to others’ results. Additionally, senior adults accounted for 62.5% (5/8) of unfavorable, much more than 26.8% (11/41) of the favorable group, although the statistic was not significant enough in the npmSAH subgroup (*p* = 0.12).

HCS was first illuminated in 1990 as a semi-quantitative scale to evaluate the SAH volume (12). This scale was found to provide better reliability and prognostic accuracy, with superior interobserver agreement, than previous grading scales (23, 24). HCS was composed of 10 basal cisterns and fissures, including frontal interhemispheric fissure; lateral parts of sylvian fissure; basal parts of sylvian fissure; suprasellar cistern; ambient cisterns and quadrigeminal cistern. The process of evaluation was also simple and rapid. Kole et al. created a Hijdra score predictive model to evaluate the occurrence of aneurysm in SAH patients (25). Bretz et al. demonstrated Hijdra score has prognostic value for the neurological outcome in SAH patients (26). Currently, we uncovered that a novel value of Hijdra score and elevation of HCS was associated with unfavorable outcome for both anSAH and npmSAH subgroup. More bleeding volume indicated more serious inflammation of vessels and more possibility for vasospasm and cerebral infarction, leading to unfavorable outcome (27, 28). Finally, we further confirmed the hypothesis by multivariate analysis and revealed that high HCS was an independent risk factor for long-term prognosis after npmSAH.

Hydrocephalus is a serious and common complication in the clinical course of SAH. According to various clinical circumstances, a wide range of incidence of hydrocephalus in SAH patients from 6% to 67% has been reported, mainly concentrated around 30% (29, 30). While hydrocephalus after anSAH is actually minimal and uncommon, even for those with a diffuse, aneurysmal-like pattern of bleeding. Blood clots blocking the cerebral aqueducts or fourth ventricle outlet, small blood clots obstructing the arachnoid villi and fibrosis within the subarachnoid space induced by hemoglobin are the reliable mechanism of post-hemorrhagic hydrocephalus (28, 31). Surgical interventions include temporary lumbar CSF drainage, LCFD and placement of a permanent ventricular shunt. Eighteen patients suffered LCFD and the meantime were 99.8 ± 32.4 h and long-time LCFD was associated with the malignant clinical course in our project (*p* = 0.029). No permanent shunt was performed in our patients, consistent with the extremely low chance of ventriculoperitoneal shunt for anSAH (6, 7). Samuels et al. reported that hydrocephalus was a risk factor for poor functional outcome in SAH patients (32), as we have demonstrated in our analysis.

The study had several limitations. First, the sample size is not large enough to draw more solid conclusions, especially for pmSAH. To be honest, we are not a huge neurological center and partially due to the naturally low incidence of this entity. Second, due to the retrospective single-center design, there are typical restrictions such as loss of follow-up, regimen changes and selection bias. In the future, we plan to design a prospective, multi-center and large-size study to further confirm our conclusion and explore the intervention for functional outcome. Third, despite HCS being evaluated by the two experienced neurologists, intraobserver variability in grading the amount of blood might have affected the accuracy of the HCS. Fourth, a univariate analysis may not fully establish a cause-and-effect relationship between unfavorable outcomes and longer CSF drainage periods as well as higher HCS scores of anSAH patients.

In conclusion, the results of the present study provide valuable information on both SAH patterns and functional outcome in patients suffering from angiography-negative non-traumatic SAH and should be taken into account during management of these patients.

## Data availability statement

The original contributions presented in the study are included in the article/supplementary material, further inquiries can be directed to the corresponding author.

## Ethics statement

The study was approved by the Beijing Chaoyang Hospital Ethics Committee. Written informed consent for participation was not required for this study in accordance with the national legislation and the institutional requirements.

## Author contributions

YG and YW contributed to conception and design of the study. JJ and XL performed the follow-up. XL and YG performed the statistical analysis. TL and HL assessed the radiological characteristics. YG and JJ wrote the first draft of the manuscript. YS and YW supervised the study. All authors contributed to the article and approved the submitted version.

## Funding

This work was supported by the “Golden Seed” Project of Beijing Chaoyang Hospital (no. CYJZ202157-21) and the National Natural Science Foundation of China (no. 81960330).

## Conflict of interest

The authors declare that the research was conducted in the absence of any commercial or financial relationships that could be construed as a potential conflict of interest.

## Publisher’s note

All claims expressed in this article are solely those of the authors and do not necessarily represent those of their affiliated organizations, or those of the publisher, the editors and the reviewers. Any product that may be evaluated in this article, or claim that may be made by its manufacturer, is not guaranteed or endorsed by the publisher.

## Footnotes

^1^ https://www.who.int/data/gho/data/themes/topics/topic-details/GHO/body-mass-index
